# Detection and sequence analysis of accessory gene regulator genes of *Staphylococcus pseudintermedius* isolates

**DOI:** 10.14202/vetworld.2015.902-907

**Published:** 2015-07-23

**Authors:** M. Ananda Chitra, C. Jayanthy, B. Nagarajan

**Affiliations:** 1 Department of Veterinary Microbiology, Madras Veterinary College, Tamil Nadu Veterinary and Animal Sciences University, Chennai - 600 007, Tamil Nadu, India; 2 Department of Veterinary Clinical Medicine, Madras Veterinary College, Tamil Nadu Veterinary and Animal Sciences University, Chennai - 600 007, Tamil Nadu, India

**Keywords:** accessory gene regulator, dog, skin infections, *Staphylococcus pseudintermedius*

## Abstract

**Background:**

*Staphylococcus pseudintermedius* (SP) is the major pathogenic species of dogs involved in a wide variety of skin and soft tissue infections. The accessory gene regulator (*agr*) locus of *Staphylococcus* aureus has been extensively studied, and it influences the expression of many virulence genes. It encodes a two-component signal transduction system that leads to down-regulation of surface proteins and up-regulation of secreted proteins during *in vitro* growth of *S. aureus*. The objective of this study was to detect and sequence analyzing the AgrA, B, and D of SP isolated from canine skin infections.

**Materials and Methods:**

In this study, we have isolated and identified SP from canine pyoderma and otitis cases by polymerase chain reaction (PCR) and confirmed by PCR-restriction fragment length polymorphism. Primers for SP *agrA* and *agrBD* genes were designed using online primer designing software and BLAST searched for its specificity. Amplification of the *agr* genes was carried out for 53 isolates of SP by PCR and sequencing of agrA, B, and D were carried out for five isolates and analyzed using DNAstar and Mega5.2 software.

**Results:**

A total of 53 (59%) SP isolates were obtained from 90 samples. 15 isolates (28%) were confirmed to be methicillin-resistant SP (MRSP) with the detection of the *mecA* gene. Accessory gene regulator A, B, and D genes were detected in all the SP isolates. Complete nucleotide sequences of the above three genes for five isolates were submitted to GenBank, and their accession numbers are from KJ133557 to KJ133571. AgrA amino acid sequence analysis showed that it is mainly made of alpha-helices and is hydrophilic in nature. AgrB is a transmembrane protein, and *AgrD* encodes the precursor of the autoinducing peptide (AIP). Sequencing of the agrD gene revealed that the 5 canine SP strains tested could be divided into three Agr specificity groups (RIPTSTGFF, KIPTSTGFF, and RIPISTGFF) based on the putative AIP produced by each strain. The AIP of SP contains serine and produce lactone ring structured AIP.

**Conclusion:**

Presence of AgrA, B, and D in all SP isolates implies the importance of this regulatory system in the virulence genes expression of the SP bacteria. SP isolates can be typed based on the AgrD auto-inducible protein sequences as it is being carried out for typing of *S. aureus* isolates. However, further studies are required to elucidate the mechanism of controlling of virulence genes by *agr* gene locus in the pathogenesis of soft tissue infection by SP.

## Introduction

*Staphylococcus pseudintermedius* (SP), a novel coagulase positive staphylococcal species described in 2005 is a normal inhabitant of the skin and mucosa and can be isolated from nasal and oral mucosa, skin of forehead, groin and anus of healthy dogs and cats. SP is an opportunistic pathogen and a leading cause of skin and ear infections in dogs [1].

SP expresses a variety of virulence genes such as hemolysins, leukotoxins, extracellular enzymes, anti-inflammatory peptides, and adhesins in a coordinated manner. Numerous regulatory systems are present in *Staphylococcus aureus* for this coordinated expression of virulence factors. In quorum sensing system, gene regulation occurs in response to a threshold concentration of extracellular signal that is reached when a sufficient population, or quorum, of bacteria, is present. Accessory gene regulator (*agr*) is a conventional quorum-sensing system present in a wide range of Gram-positive and negative bacteria.

The *agr* locus of *S. aureus* has been studied extensively, and it consists of two divergent operons driven by the P2 and P3 promoters. The P2 operon contains *agrBDCA* and codes for the RNAII transcript. P3 drives transcription of RNAIII, which is the effector molecule of the Agr locus [2]. Delta-hemolysin, a secreted virulence factor encoded by *hld*, is translated from RNAIII [3]. AgrA and AgrC comprise a two-component regulatory system that responds to the secreted autoinducing cyclic octapeptide [4]. The autoinducing cyclic octapeptide is processed from the *agrD* product by AgrB. In batch culture, RNAIII acts to increase the expression of secreted virulence factors and decrease the expression of several surface ­adhesins, including protein A and the fibronectin-binding protein [5].

The objective of this study was to detect and analyze *agr* genes of SP clinical isolates from skin infections of dogs in Chennai, India.

## Materials and Methods

### Ethical approval

No ethical approval was necessary for this study; however, we obtained informed consent from all the pet owners involved in this study for sample collection and we maintained the confidentiality of the diagnostic results.

### Bacterial isolates

A sterile cotton swab was used to sample canine skin infection cases brought to Dermatology Unit of Madras Veterinary College Teaching Hospital from Feb 2013 to Feb 2014. Swabs were inoculated by streaking on mannitol salt agar plates. A single representative colony of each sample was propagated by streaking it on a nutrient agar plate and subjected to Gram’s staining, catalase tests, and other biochemical tests.

### DNA extraction

Four to five colonies were suspended in PBS and centrifuged at 6000 rpm for 10 min. The pellet was suspended in 100 µl of sterile distilled water and boiled at 100°C for 10 min. Then the tubes were cooled immediately by placing them on the ice. Later, they were centrifuged at 10000 rpm for 10 min, and the supernatant was used as template in the amplification reaction.

### Species identification by polymerase chain reaction (PCR)

PCR was carried out using the designed primers (Table-1) targeting the nuclease (*nuc*) gene of SP to differentiate *S. intermedius* groups (SIG) from *S. aureus*. PCR was performed in a reaction volume of 10 µl containing approximately 100 ng of genomic DNA, 5 pmol of each primer, and 2 × master mix (Ampliqon, Denmark). Cycling conditions were 94°C for 3 min, followed by 30 cycles of denaturation at 94°C for 30 s, annealing at 60°C for 30 s, extension at 72°C for 30 s, and a final extension cycle of 5 min at 72°C. PCR products were loaded on a 1.5% agarose gel for electrophoresis, visualized with ethidium ­bromide, and documented. Further, all the positive isolates were confirmed as SP by PCR-restriction fragment length polymorphism (RFLP) targeting phospho acetyltransferase gene sequence that was developed by Bannoehr *et al*. [6].

**Table-1:** Primers used in this study along with amplicon size, annealing temperature, and references.

Serial number	Primer name	Sequences	Amplicon size (bp)	Annealing temperature	Reference
1	NUC	F5’ AAACACCGAGTAATACGCCG 3’ R 5’TTTAGCGTTCCCAAATGTTCAG3’	780	60°C	This study
2	PTA	F5’ AAAGACAAACTTTCAGGTAA3’ R5’ GCATAAACAAGCATTGTACCG3’	320	55°C	Bannoehr *et al.* (2009)
3	AGR-A	F5’ CCTATCCGGGAAATTGGCTTT 3’ R 5’ CGGACAATGTATTCCTTGATACGA3’	850	60°C	This study
4	AGR-BD	F5’ GGATGAGAATAATGTAATCCCTTTGAC3’ R 5’ AACAACCAATCACAACAGTTAGG 3’	946	60°C	This study
5	MecA	F 5’- CAAACTACGGTAACATTGATCGC-3’ R 5’- GCCTATCTCATATGCTGTTCCT-3’	210	60°C	This study

### Detection of agr genes and DNA sequencing analysis

Primers for *agrA* and *agrBD* were designed using online idt primer quest™ software of Integrated DNA Technologies, USA (www.idtdna.com) using SP ED99 strain and HKU10-03 strain *agr* gene sequences available in the NCBI database (Accession No. CP002478 and CP002439, respectively). Then, the selected primers were BLAST searched for the specificity using *Blastn* program of NCBI. Amplification of *agr* genes were performed as mentioned above, and purification of the amplicon was done using PCR purification kit (Real Biotech Corporation, Taiwan) as per the manufacturer’s instruction. DNA sequencing of purified nucleotides was done with automated high throughput nucleic acid sequence of Applied Biosystems 3500 Instrument model. Homology searches were performed with the NCBI database and BLAST. Alignment and phylogenetic tree analysis were carried out using the Mega5.2 software. *In silico* amino acid analysis was done using *DNAstar* software.

### Nucleotide sequence accession number

The GenBank accession numbers for the nucleotide sequence of *agrA, agrB*, and *agrC* genes of five SP isolates are from KJ133557 to KJ133571.

## Results and Discussion

PCR targeting SP thermonuclease gene yielded 780 bp amplicon in 53 staphylococcal isolates (59%) of 90 samples. This PCR had been a useful tool in the preliminary identification of SP and it also useful to differentiate SP isolates from other coagulase positive staphylococcal species such as *S. aureus* and *Staphylococcus intermedius* organisms. All the staphylococcal isolates were subjected to PCR-RFLP targeting phospho acetyltransferase sequence for further confirmation as SP. SP contained a single *Mbo*I site, resulting in two fragments of 213 bp and 107 bp whereas other staphylococcal isolates did not contain an *Mbo*I restriction site and appeared as a single undigested band of 320 bp. Of the 53 SP isolates, 24 were from pyoderma, 10 were associated with demodicosis, 5 from otitis cases, and 13 were from others cases such as skin allergy, tick infestations, etc. SP was the predominant isolate of canine skin infections in the present study and this is in accordance with the number of other studies [7-11].

Methicillin-resistant SP (MRSP) has emerged as a significant pet animal health problem in veterinary medicine and resistant is mediated by *mecA* gene that encodes production of a modified penicillin-binding protein. In this study, designed *mecA* gene primers amplified a product of 210 bp size, and it was significantly detected in 15 (28%) SP isolates. To the best of our knowledge, a previous report on the prevalence of MRSP from India is not available for comparative studies. MRSP was first reported in 2005 and since then more numbers of MRSP were isolated from various countries. In this study, the frequency of MRSP is 28% which is comparatively lesser than the prevalence of MRSP in western countries [11], Japan [12], and China [13] but significantly higher than the occurrence of MRSP in West Indies [14] and Croatia [15].

**Figure-1:**
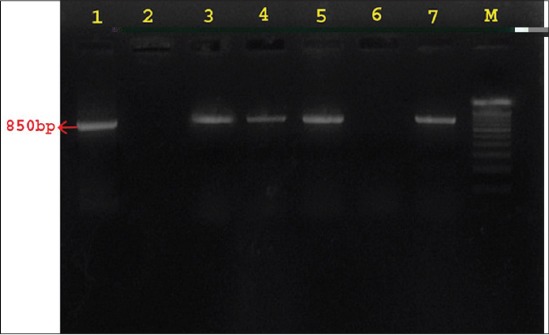
Agarose gel electrophoresis showing the results of PCR amplified product of 850 bp for the *agrA* gene of *Staphylococcus pseudintermedius* (SP) isolates. Lane 1, 3, 4, 5, 7: *agrA* gene positive for SP isolates; Lane 2 and 6: *Staphylococcus aureus* isolates negative for *agrA* gene of SP; Lane M: 100 bp DNA ladder.

**Figure-2:**
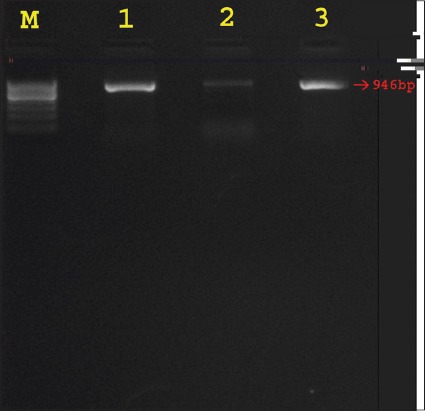
Agarose gel electrophoresis showing the results of polymerase chain reaction amplified product of 946 bp for the *agrBD* gene of *Staphylococcus pseudintermedius* isolates. Lane M: 100 bp DNA ladder; Lane 1, 2 and 3: *agrBD* gene positive for *S. pseudintermedius* isolates.

Amplification with *agrA* primers yielded expected amplicon of 850bp (Figure-1) and with agrBD primers gave sharp band size of 946bp (Figure-2). Accessory gene regulator A, B, and D genes were detected in all SP isolates which imply the importance of this regulatory system in the virulence genes expression of the SP bacteria. Amino acid sequence of AgrA, AgrB, and AgrD were BLAST searched for sequence similarities and found that they share some sequence similarity with *S. aureus*, *Staphylococcus epidermidis*, *Staphylococcus Lugdunensis*, and more than 95% sequence similarities with *S. intermedius* and *Staphylococcus delphini*. Among the three Agr sequences, AgrA sequences revealed that they are highly conserved than AgrB and AgrD in all isolates as well as with the available genome sequences of SP in GenBank, NCBI.

**Figure-3:**
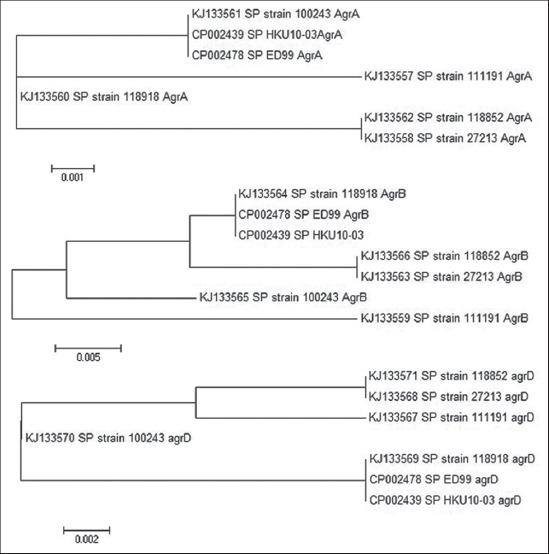
(a-c) Phylogenetic tree based on AgrA, AgrB, and AgrD amino acid sequences of *Staphylococcus pseudintermedius* isolates.

AgrA amino acid sequence analysis showed that it is made of mainly alpha-helices with turns and few beta-pleated sheets in the C-terminal end. Phylogenetic relationship of AgrA sequences of SP Chennai isolates with available SP organisms in GenBank, NCBI was given in Figure-3a. AgrA sequences of SP Chennai isolate 100243 was more closely related to European SP isolates than the other Chennai isolates. AgrA of *S. aureus* is a member of a family of conserved transcriptional response regulators which contains a regulatory domain and unique LytTR family of DNA-binding domain [16]. These response regulators undergo conformational changes upon the phosphorylation of an aspartate residue by the cognate sensory histidine kinase (AgrC), allowing them to bind to promoter elements and up-regulate transcription. Activated AgrA dimerizes, binds either to strong P2 or weak P3 promoter site and transcribes the regulatory RNAIII, ultimately activating virulence gene expression [17]. Traber and Novick [18] identified that a mutation in AgrA of a laboratory strain of *Staphylococcus* resulted in the delayed activation of *agr* system and failed to produce effectors molecules (hemolysins) when compared to clinical isolates. There is a frame-shift “slip” whereby AgrA is lengthened at the C-terminus by 3 or 21 residues - the former slows AgrA activation and the latter abolishes it altogether.

AgrB amino acid sequence analysis showed that it comprises a combination of alpha-helices and beta pleated sheets with more hydrophobicity and less surface probability. Structure and function analysis of AgrB of *S. aureus* is a well-studied model compared with AgrB of other staphylococcal species. AgrB of *S. aureus* is a 22 kDa transmembrane protein, which is responsible for processing AgrD into the final autoinducing peptide (AIP) product [19]. Many studies in *S. aureus* have demonstrated that AgrB has endopeptidase activity that can remove the AgrD C-terminal tail to produce AIP. Two residues, histidine-77 and cysteine-84 were identified as being essential for the AgrB proteolytic activity, suggesting that AgrB acts as a cysteine protease [20,21]. These two residues are absolutely conserved in all five isolates of SP, highlighting their importance in AgrB function. AgrB of staphylococcal *agr* system is unique in nature because of the lack of sequence similarity with other cysteine proteases or any other quorum-sensing proteins [2]. Phylogenetic relationship of AgrB sequences of SP Chennai isolates with available SP isolates sequences of GenBank, NCBI was given in Figure-3b. AgrB sequences of SP Chennai isolate 118918 was closely segregated with the European isolates sequences.

The AgrD propeptide consists of 45 amino acids, and the middle section of AgrD encodes the final nine residues AIP-molecule as shown in Table-2 in detail. The putative AIP of SP contains nonapeptide whereas in *S. aureus* it may vary from 8 to 9 peptides. The notable feature of AIP of SP is that it contains serine at 5^th^ position and hence produce lactone ring structured AIP. In contrast, *S. aureus* AIP contains cysteine in 5^th^ position from carboxyl terminal and produces an AIP containing a thiolactone ring structure [22].

**Table-2:** Details of SP isolates, sequences of AgrD, and agr types.

Strain number	*mec*A	SpA	N-terminal AgrD	AIP	C-terminal AgrD	Agr type
118852	-	+	MRILEVLFNLITNLFQSIGTFA	RIPTSTGFF	DEPEIPAELLEEDK	I
D27213	-	+	MRILEVLFNLITNLFQSIGTFA	RIPTSTGFF	DEPEIPAELLEEDK	I
111191	-	+	MRILEVLFNLITNLFQSIGTFA	RIPISTGFF	DEPEIPAELLEEDK	II
118918	+	+	MRILEVLFNLITNLFQSIGTFA	KIPTSTGFF	DEPEIPAELLEEDK	III
100243	-	-	MRILEVLFNLITNLFQSIGTFA	KIPTSTGFF	DEPEIPEELLEEDK	III
SP HKU10-03	-	-	MRILEVLFNLITNLFQSIGTFA	KIPTSTGFF	DEPEIPEELLEEDK	III
SP ED99	-	+	MRILEVLFNLITNLFQSIGTFA	KIPTSTGFF	DEPEIPEELLEEDK	III

SP=*Staphylococcus pseudintermedius*

Sequence analyses of the *agrD* genes of five strains of SP clinical isolates revealed that they could be typed into three AIP allelic variants. Among the 5 strains, one strain produced AIP of RIPISTGFF while RIPTSTGFF and KIPTSTGFF AIP were produced by 2 strains of each. It had been found that there was no significant correlation on the susceptibility to methicillin, the expression of staphylococcal protein A and Agr types. Phylogenetic relationship of AgrD sequences of SP Chennai isolates with available SP isolates sequences of GenBank, NCBI was given in Figure-3c. AgrD sequences of SP Chennai isolate 118918 was segregated with the European SP isolates HKU10-03 and ED99, and AIP sequences of all three were Type III KIPTSTGFF.

Sequence analysis of *agrD* genes of SP collected from pyoderma cases by Sung *et al*. [23] also revealed the distribution of three AIP variants. In their study, among the 20 strains, 5% (1 strain) produced the AIP RIPTSTGFF, while 35% (7 strains) and 60% (12 strains) produced the RIPISTGFF and KIPTSTGFF peptides, respectively. Bannoehr *et al*. [7] reported the fourth AIP variant KYPTSTGFF in 27% of the total 104 SP strains. AgrD sequence analysis of more numbers of SP isolates is required to know the status of the presence of this fourth SP AIP in this geographical part of the world.

The sequence of AgrD was BLAST searched for homology and revealed that this gene is highly conserved among the members of SIG. The C-terminal tail is the most conserved portion of AgrD among staphylococcal species, especially the first six residues. The tail always starts with aspartate and glutamate as the first two amino acids followed by three other absolutely conserved Proline, Glutamate, and Leucine residues at the sixth, eighth, and ninth positions. In the C-terminus of AgrD propeptide of one SP isolate, Alanine at 7^th^ position is replaced with Glutamate. The negatively charged C-terminus has been proposed to be involved in the processing of AgrD and it contributes to the translocation of the AIP [24].

The four *agr*-specific groups were identified in *S. aureus* on the basis of the specificity of the autoinducer receptor, AgrD-AgrC [25]. It has been reported that *agr* group IV strains were associated with generalized exfoliative syndromes, *agr* group I strains involved in invasive infections, especially bacteremia and *agr* Groups I and II strains causing mainly endocarditis, and TSS toxin 1-producing isolates belong to *agr* specificity Group III [26]. The detailed studies of such association of Agr types and disease it causes are not characterized for SP bacterial isolates and are highly warranted at present.

## Conclusion

Presence of AgrA, B, and D in all SP isolates implies the importance of this regulatory system in the virulence genes expression of the SP bacteria. SP isolates can be typed based on the AgrD auto-inducible protein sequences as it is being carried out for typing of *S. aureus* isolates. As far as our knowledge is concerned that this is the first report on the detection and characterization of agr A, B, and D of SP isolates from India. More researches have been carried out on the Agr system of *S. aureus* whereas further studies are required to elucidate the mechanism of controlling of virulence genes by *agr* gene locus in the pathogenesis of soft tissue infection by SP.

## Authors’ Contributions

MAC has designed and carried out the laboratory work, analyzed and compiled results, and also prepared the manuscript. CJ and BN have equally contributed in the collection of samples and in final editing. All authors read and approved the final manuscript.
